# An Extension of Testlet-Based Equating to the Polytomous Testlet Response Theory Model

**DOI:** 10.3389/fpsyg.2021.743362

**Published:** 2022-01-12

**Authors:** Feifei Huang, Zhe Li, Ying Liu, Jingan Su, Li Yin, Minqiang Zhang

**Affiliations:** ^1^School of Psychology, South China Normal University, Guangzhou, China; ^2^College of Teacher’s Education, Guangdong University of Education, Guangzhou, China

**Keywords:** testlet, test equating, item response theory model, dichotomous testlet response theory model, polytomous testlet response theory model

## Abstract

Educational assessments tests are often constructed using testlets because of the flexibility to test various aspects of the cognitive activities and broad content sampling. However, the violation of the local item independence assumption is inevitable when tests are built using testlet items. In this study, simulations are conducted to evaluate the performance of item response theory models and testlet response theory models for both the dichotomous and polytomous items in the context of equating tests composed of testlets. We also examine the impact of testlet effect, length of testlet items, and sample size on estimating item and person parameters. The results show that more accurate performance of testlet response theory models over item response theory models was consistently observed across the studies, which supports the benefits of using the testlet response theory models in equating for tests composed of testlets. Further, results of the study indicate that when sample size is large, item response theory models performed similarly to testlet response theory models across all studies.

## Introduction

In the current practice of educational measurement, test equating is a vital step to put scores from different forms onto a same scale. However, in most large-scale testing programs, it is common for a standardized test to consist of testlets (Bradlow et al., 1999; Rijmen, 2009; Cao et al., 2014; Tao and Cao, 2016). A testlet is defined as an aggregation of items which are based on a common stimulus (Wainer and Kiely, 1987; Bradlow et al., 1999). Responses to items within a testlet often tend to violate the local item independence. For example, some examinees that are more familiar with the background information covered by the testlet may have a higher probability to correctly answer the items of a specific testlet (Rijmen, 2009; Cao et al., 2014; Tao and Cao, 2016). Although researchers have conducted an abundance of studies to propose different approaches to handle local item dependence (LID), little research in the literature has focused on the performance of different approaches to accommodate LID on testlet-based test equating.

Studies have shown that the accuracy of parameter estimation produced by the testlet response theory (TRT) model is higher than the traditional item response theory (IRT) model where LID was present (Bradlow et al., 1999; Wainer and Wang, 2000; Wainer et al., 2000; Zhang, 2010; Koziol, 2016). However, numbers of studies were based on dichotomous items (Wainer and Wang, 2000; Rijmen, 2009; Cao et al., 2014). Researchers have found that although the polytomous IRT models suffer the problem of losing response pattern information, they are still much easier in interpretation and implementation (Sireci et al., 1991; Zenisky et al., 2002; Cao et al., 2014). Moreover, studies have also documented that the dichotomous IRT models could lead to misestimation of test reliability and item parameters (Sireci et al., 1991; Lawrence, 1995; Zenisky et al., 2002; Keller et al., 2003; Cao et al., 2014). Because there is little evidence about the application of TRT models for the polytomous items composed of testlets in the context of equating tests, it is not clear how the performance of TRT models might be.

It is needed to place the IRT estimates from different test forms on a common scale when conducting test equating (Kolen and Brennan, 2014). Generally, there are two kinds of parameter linking methods known as separate calibration and concurrent calibration (von Davier and von Davier, 2011; Kolen and Brennan, 2014; González and Wiberg, 2017). Separate calibration needs an equating transformation to perform the equating, while concurrent calibration can link parameters obtained from different test forms on a common scale during the estimation routine (Kolen and Brennan, 2014; González and Wiberg, 2017). Researches of test equating on the testlets were mostly based on the method of separate calibration (Lee et al., 2001; Cao et al., 2014; Tao and Cao, 2016). However, studies have shown that the accuracy of equating results of concurrent calibration were higher (Wingersky et al., 1987; Hanson and Beguin, 2002). Further, the method of concurrent calibration is easy in implementation.

Studies have investigated the influence of testlet effect size on test reliability and parameter estimates produced by IRT and TRT models (Bradlow et al., 1999; Wang et al., 2002; Zhang, 2010; Cao et al., 2014; Koziol, 2016). In addition to the testlet effect size, few studies have simultaneously investigated the impact of length of testlet items and sample size on the testlet models. However, sample size and the length of testlet items are also the important factors which can affect the accuracy of parameter estimation and equating results (Tao and Cao, 2016). Therefore, it is vital to consider those factors to compare the performance of IRT models and TRT models.

## Testlet Response Theory Models

Bradlow et al. (1999) first proposed a dichotomous testlet item response model, which is based on the two-parameter logistic model (2PLM) and incorporated the random item testlet effect parameter. Since then, the TRT models have been introduced in a series of papers (Wainer et al., 2000, 2007; Wainer and Wang, 2001; Wang et al., 2002, 2004; Wang and Wilson, 2005). Researchers have found that the TRT models are predominantly used to represent the multidimensional IRT approach to model LID due to testlet effects (DeMars, 2006; Li et al., 2006).

The 2PLM and the two-parameter TRT model (2PTM) can be expressed as:


(1) $$\text{P}\left(y_{i\,j}=1\right)=\frac{\text{exp}\,\left[a_{j}\,\left(\theta_{i}-b_{j}\right)\right]}{1+\text{exp}\,\left[a_{j}\,\left(\theta_{i}-b_{j}\right)\right]}$$

(2) $$\text{P}\left(y_{i\,j}=1\right)=\frac{\text{exp}\,\left[a_{j}\,\left(\theta_{i}-b_{j}-\gamma_{i\,d\,\left(j\right)}\right)\right]}{1+\text{exp}\,\left[a_{j}\,\left(\theta_{i}-b_{j}-\gamma_{i\,d\,\left(j\right)}\right)\right]},$$

where *a*_*j*_ is the discrimination parameter for item *j*, *b*_*j*_ is the difficulty parameter for item *j*, θ*_*i*_* is the latent trait level for examinee *i*. For the TRT model, *d*(*j*) denotes a testlet containing item *j*, γ_*id*(*j*)_ is the random effect for examinee *i* on testlet *d*(*j*), which describes the interaction between examinee’s performance on the testlet and items (LID) within the testlet. The model assumes that$\gamma_{i\,d\,\left(j\right)}∼N[0,\sigma_{\gamma_{i\,d\,\left(j\right)}}^{2}$]. Note that$\sigma_{\gamma_{i\,d\,\left(j\right)}}^{2}$ reflects the amount of the testlet effect. The larger the $\sigma_{\gamma_{i\,d\,\left(j\right)}}^{2}$is, the larger the testlet effect will be.

However, the increasing number of educational tests consisted of polytomous item have received a substantial amount of attention because of the need for more realistic and richer forms of assessment. Therefore, researchers extended the graded response model (GRM) which is widely used to a graded response testlet model (Wang et al., 2002). Further, they developed the corresponding software SCORIGHT3.0 to estimate parameters by using the Monte Carlo method within the Bayesian framework (Wang et al., 2004).

The GRM and graded response testlet model (GRTM) can be expressed as:


(3) $$P_{m\,x}^{*}\left(\theta \right)=\frac{\exp\left[\alpha_{m}\,\left(\theta_{i}-b_{m\,x}\right)\right]}{1+\exp\left[\alpha_{m}\,\left(\theta_{i}-b_{m\,x}\right)\right]}\left(x=0,1,2,\ldots ,k_{m}-1\right)$$

(4) $$P_{m\,x}\,\left(\theta \right)=P_{m\,x}^{*}\,\left(\theta \right)-P_{m\,\left(x+1\right)}^{*}\,\left(\theta \right)$$

(5) $$P_{j\,n}^{*}\left(\theta \right)=\frac{\exp\left[\alpha_{j}\,\left(\theta_{i}-b_{j\,n}-\gamma_{i\,d\,\left(j\right)}\right)\right]}{1+\exp\left[\alpha_{j}\,\left(\theta_{i}-b_{j\,n}-\gamma_{i\,d\,\left(j\right)}\right)\right]}\left(n=0,1,2,\ldots ,k_{j}-1\right)$$

(6) $$P_{j\,n}\,\left(\theta \right)=P_{j\,n}^{*}\,\left(\theta \right)-P_{j\,\left(n+1\right)}^{*}\,\left(\theta \right),$$

where$P_{m\,x}^{*}\,\left(\theta \right)$ is the probability of an examinee with a given θ responding to category *x* or higher of item *m*, *a*_*m*_ is the discrimination parameter for item *m*, *b*_*mx*_ is the category boundary for score *x* on item *m*, *P*_*mx*_(θ) is the probability of an examinee with a given θ will score in a particular category of item *m*. Compared with the GRM, *d*(*j*) denotes a testlet containing item *j*, γ_*id*(*j*)_ is the random effect for examinee *i* on testlet *d*(*j*).

### The Present Study

This paper presents the results of two simulation studies that addresses these two issues. First, the performance of IRT models and TRT models for the dichotomous and polytomous items by using the concurrent calibration in the context of equating tests composed of testlets was assessed. Second, the effect of testlet effect, sample size and length of testlet items on parameter estimates produced by IRT and TRT models was investigated.

The rest of this article is organized as follows. First, the IRT and TRT models for the dichotomous and polytomous items are briefly introduced. Second, two simulation studies are conducted to assess the IRT models and TRT models. These simulations also demonstrate how testlet effect, length of testlet items, and sample size affect item and person parameters estimation. Finally, this article draws conclusions for the performance of IRT models and TRT models and suggestions for future study are provided.

## Materials and Methods

### Study Design

The simulation study employed the non-equivalent anchor test (NEAT) design. In the NEAT design, two simulations with several manipulated factors were conducted to compare the performance of item response models and testlet response models in the context of equating tests composed of testlets (as shown in Table 1). For the first simulation study, four major independent variables were manipulated: (a) models (2PLM and 2PTM), (b) testlet effect (0.5, 1, and 2), (c) length of testlet items (5 and 10), and (d) sample size (1,000 and 2,000 examinees). The manipulated factors for the second simulation study were as same as the first one, except for the models. As the purpose of the second simulation study was to compare the performance of polytomous item response models and testlet response models, the GRM and the GRTM were selected.

**TABLE 1:** Summary of the study design for the two simulation studies.

	Manipulated factors			
The first simulation study	Models	2PLM	2PTM	
	Testlet effect	0.5	1	2
	Length of testlet items	5	10	
	Sample size	1,000	2,000	
The second simulation study	Models	GRM	GRTM	
	Testlet effect	0.5	1	2
	Length of testlet items	5	10	
	Sample size	1,000	2,000	

### Simulation Process

Six pairs of test forms were created with varying degree of testlet effect and different length of testlet items for each simulation research. Each test pair was consisted of a base form and a new form. Each test form had a total of 60 multiple choice items in the first simulation study or 60 polytomous items in the second simulation study, composed of 40 non-anchor items and 20 anchor items. For the non-anchor items, there were 20 locally independent items, and 4 testlets with 5 items per testlet or 2 testlets with 10 items per testlet. For the anchor items, there were 2 testlets with 5 items per testlet or 1 testlets with 10 items per testlet.

Item parameters for the base form and the new form composed solely of locally independent non-anchor items were randomly selected from the same population distributions. Specifically, ln*a* ∼ *N* (0, 1), constrained to (0, 2.5); *b* ∼ *N* (0, 1), constrained to (-3, +3). Item parameters of independent anchor items which were shared by the base form and the new form were also randomly selected from the same population distributions. Specifically, ln*a* ∼ *N* (0, 1), constrained to (1, 2); *b* ∼ *N* (0, 1), constrained to (-3, +3). The population distribution to the *a*-parameter for the anchor items with a slightly higher than the non-anchor items is to assure the representative of the anchor items (Wang et al., 2002; Cao et al., 2014; Tao and Cao, 2016). The ability population distribution for the base form is the same as the *b*-parameter population distribution to assure that the difficulty of test is appropriate for the examinees (Tao and Cao, 2016). The ability population distribution for the new form with a slightly higher mean than the base form to reflect ability differences between two groups (Lee et al., 2016; Andersson, 2018). For the three pairs of test forms, the testlet effect indexed by the $\sigma_{\gamma_{i\,d\,\left(j\right)}}^{2}$for the base form and the reference form were drawn from three uniform distributions: (0.1, 0.5), (0.6, 1), and (1.1, 2.0) corresponding to low, moderate and high levels of LID, respectively (Wang et al., 2002; DeMars, 2006; Cao et al., 2014; Tao and Cao, 2016).

The probability of each examinee’s response to each item was calculated based on the simulated parameters mentioned above using the 2PL model, the 2PTM, the GRM, and the GRTM, respectively. Then, the probability was compared with a number randomly drawn from U (0, 1). For the dichotomous items, if the probability was larger than the random number, the response was coded as “1”; otherwise, as “0.” For the polytomous items, if the random number was larger than the cumulative probability with category 1, the response was coded as “0”; if the random number was between the cumulative probability with category 1 and the cumulative probability with category 2, the response was coded as “1”; if the random number was between the cumulative probability with category 2 and the cumulative probability with category 3, the response was coded as “2”; if the random number was between the cumulative probability with category 3 and the cumulative probability with category 4, the response was coded as “3”; otherwise, as “4.”

An R (version 3.3.1, R Core Team, 2016) program was written to generate data and calibrate the response data by the 2PL model, the 2PTM, the GRM, and the GRTM, respectively. The program flexMIRT (Cai, 2017) were used to conduct the concurrent calibration. Related R codes could be requested from the correspondence author.

### Evaluation Criteria

The focus of our study was not only on comparing IRT and TRT models, but also on the effect of testlet effect, sample size and length of testlet items on parameter estimates produced by IRT and TRT models. Therefore, we used the equating bias, standard error of equating and root mean square error to assess the performance of IRT models and TRT models for the dichotomous and polytomous items in the context of equating tests composed of testlets. Bias is an indicator of systematic error in equating. SEE is an indicator of random sampling error in equating. RMSE represents the total error in equating, which were defined as


(7) $$B\,i\,a\,s=\frac{1}{R}\,\sum_{r=1}^{R}\hat{\lambda }-\lambda$$

(8) $$S\,E\,E=\sqrt{\frac{1}{R}\,\sum_{r=1}^{R}{\left(\hat{\lambda }-\bar{\hat{\lambda }}\right)}^{2}}$$

(9) $$R\,M\,S\,E=\sqrt{\frac{1}{R}\,\sum_{r=1}^{R}{\left(\hat{\lambda }-\lambda \right)}^{2}},$$

where $\hat{\lambda }$ and λ were the estimated and true values for item parameters and ability parameter, *R* was the total number of replications (Each condition was replicated 500 times in this study), and $\bar{\hat{\lambda }}$ was the average of $\hat{\lambda }$over the *R* replications.

## Results

Tables 2, 3 summarize the results of computing the RMSE, bias and SEE of equating accuracy of the discrimination parameter and difficulty parameter for the dichotomous testlet item. In terms of the bias and SEE, it is clear that the values of 2PTM were smaller than that of 2PLM. The discrimination parameters were overestimated for all conditions, but the difficulty parameters were underestimated. With regard to the RMSE, the RMSE values of 2PTM were smaller than that of 2PLM across all simulation conditions. Besides, a large sample size resulted in a smaller bias and RMSE. The bias, SEE and RMSE of 2PLM increased as the testlet effect and the length of testlet increased. However, no systematic patterns were observed for the bias, SEE and RMSE of 2PTM as the testlet effect and the length of testlet increased. In summary, the 2PTM had higher equating accuracy than the 2PLM for the discrimination parameter and difficulty parameter under different simulation conditions. Further, both two models could reduce the equating error with a larger sample.

**TABLE 2:** Statistical summary of the discrimination parameter for the dichotomous testlet items.

Evaluation criteria	Model	Sample size	Length of testlet = 5			Length of testlet = 10		
			Testlet effect					
			0.5	1	2	0.5	1	2
RMSE	2PLM	1,000	0.51	0.73	1.22	0.56	0.80	1.24
		2,000	0.46	0.63	0.92	0.50	0.76	1.12
	2PTM	1,000	0.35	0.43	0.46	0.36	0.45	0.49
		2,000	0.32	0.40	0.42	0.29	0.43	0.47
Bias	2PLM	1,000	0.42	0.58	0.97	0.46	0.72	1.07
		2,000	0.39	0.53	0.91	0.41	0.70	1.02
	2PTM	1,000	0.20	0.25	0.31	0.21	0.30	0.27
		2,000	0.19	0.23	0.28	0.18	0.25	0.28
SEE	2PLM	1,000	0.29	0.44	0.74	0.32	0.35	0.63
		2,000	0.24	0.34	0.14	0.29	0.30	0.46
	2PTM	1,000	0.29	0.35	0.34	0.29	0.34	0.41
		2,000	0.26	0.33	0.31	0.23	0.35	0.38

**TABLE 3:** Statistical summary of the difficulty parameter for the dichotomous testlet items.

Evaluation criteria	Model	Sample size	Length of testlet = 5			Length of testlet = 10		
			Testlet effect					
			0.5	1	2	0.5	1	2
RMSE	2PLM	1,000	0.22	0.23	0.29	0.23	0.27	0.34
		2,000	0.21	0.22	0.28	0.20	0.23	0.30
	2PTM	1,000	0.21	0.13	0.16	0.14	0.14	0.17
		2,000	0.12	0.11	0.13	0.12	0.13	0.16
Bias	2PLM	1,000	−0.10	−0.12	−0.15	−0.11	−0.14	−0.18
		2,000	−0.09	−0.10	−0.13	−0.10	−0.13	−0.16
	2PTM	1,000	−0.10	−0.08	−0.07	−0.08	−0.08	−0.07
		2,000	−0.07	−0.07	−0.06	−0.07	−0.06	−0.06
SEE	2PLM	1,000	0.20	0.20	0.25	0.20	0.23	0.29
		2,000	0.19	0.20	0.25	0.17	0.19	0.25
	2PTM	1,000	0.20	0.20	0.25	0.20	0.23	0.29
		2,000	0.10	0.08	0.12	0.10	0.12	0.15

The values of RMSE, bias and SEE of the ability parameter for the dichotomous testlet item across all simulation conditions are presented in Table 4. In terms of the bias, the ability parameter was underestimated under different conditions. A short length of testlet and a small testlet effect were associated with a more precise estimation of the ability parameter. In addition, similar trends can also be observed that the RMSE, bias and SEE decreased as the sample size increased. On the whole, the 2PTM performed better than the 2PLM.

**TABLE 4:** Statistical summary of the ability parameter for the dichotomous testlet items.

Evaluation criteria	Model	Sample size	Length of testlet = 5			Length of testlet = 10		
			Testlet effect					
			0.5	1	2	0.5	1	2
RMSE	2PLM	1,000	0.24	0.27	0.31	0.26	0.31	0.37
		2,000	0.22	0.26	0.27	0.24	0.29	0.34
	2PTM	1,000	0.18	0.21	0.23	0.20	0.21	0.27
		2,000	0.17	0.19	0.22	0.18	0.20	0.24
Bias	2PLM	1,000	−0.11	−0.12	0.14	−0.13	−0.15	−0.19
		2,000	−0.10	−0.11	0.12	−0.11	−0.14	−0.15
	2PTM	1,000	−0.08	−0.08	0.07	−0.07	−0.08	−0.07
		2,000	−0.07	−0.06	0.06	−0.06	−0.07	−0.06
SEE	2PLM	1,000	0.21	0.24	0.28	0.23	0.27	0.32
		2,000	0.20	0.24	0.24	0.21	0.25	0.31
	2PTM	1,000	0.16	0.19	0.22	0.19	0.19	0.26
		2,000	0.15	0.18	0.21	0.17	0.19	0.23

Table 5 summarizes the results of computing the RMSE, bias and SEE of equating accuracy of the discrimination parameter for the polytomous testlet item. In terms of the bias and SEE, it is clear that the values of GRTM were smaller than that of GRM. The discrimination parameters were overestimated for all conditions. With regard to the RMSE, the RMSE values of GRTM were smaller than that of GRM across all simulation conditions. The same findings for the dichotomous testlet item applied to the polytomous testlet item, as evidenced by the results that a long length of testlet (i.e., 10) and a high testlet effect (i.e., 2) resulted in a larger RMSE of GRTM. In addition, the patterns of the bias, SEE and RMSE of GRTM were the same as those in the dichotomous testlet item. Additionally, a large sample size resulted in a smaller bias and RMSE for both GRM and GRTM.

**TABLE 5:** Statistical summary of the discrimination parameter for the polytomous testlet items.

Evaluation criteria	Model	Sample size	Length of testlet = 5			Length of testlet = 10		
			Testlet effect					
			0.5	1	2	0.5	1	2
RMSE	GRM	1,000	0.65	0.69	0.97	0.67	0.72	1.04
		2,000	0.61	0.63	0.91	0.67	0.70	0.95
	GRTM	1,000	0.38	0.37	0.32	0.32	0.33	0.29
		2,000	0.36	0.34	0.31	0.31	0.32	0.29
Bias	GRM	1,000	0.69	0.74	1.03	0.73	0.83	1.12
		2,000	0.68	0.73	1.02	0.71	0.79	1.04
	GRTM	1,000	0.41	0.43	0.39	0.34	0.36	0.45
		2,000	0.38	0.37	0.39	0.32	0.35	0.42
SEE	GRM	1,000	0.23	0.27	0.35	0.29	0.41	0.42
		2,000	0.30	0.37	0.46	0.23	0.37	0.42
	GRTM	1,000	0.15	0.22	0.22	0.11	0.14	0.34
		2,000	0.12	0.15	0.24	0.08	0.14	0.30

Regarding the difficulty parameter for the polytomous testlet item, as shown in Table 6, with regard to the bias, SEE and RMSE, a long testlet length was associated with a less precise estimation of the difficulty parameter, and testlet effect and sample size had a trivial impact on the difficulty parameter for the GRTM. Additionally, the results were consistent across all categories. On the contrary, the difficulty parameter estimation was worse with a longer testlet length and a larger testlet effect for the GRM. Furthermore, the difficulty parameter estimation of the category 1 and category 4 were more deteriorated compared with the category 2 and category 3 for the GRM. Similarly, the sample size had a trivial effect on the on the difficulty parameter for the GRM. In summary, the GRTM performed better than the GRM.

**TABLE 6:** Statistical summary of the difficulty parameter for the polytomous testlet items.

Evaluation criteria	Model	Sample size	Length of testlet	Testlet effect											
				0.5				1				2			
				*b*_1_	*b*_2_	*b*_3_	*b*_4_	*b*_1_	*b*_2_	*b*_3_	*b*_4_	*b*_1_	*b*_2_	*b*_3_	*b*_4_
*RMSE*	GRM	1,000	5	0.35	0.20	0.24	0.43	0.36	0.21	0.25	0.45	0.41	0.23	0.28	0.49
			10	3.49	1.89	1.74	3.22	3.21	1.75	1.61	2.98	2.99	1.61	1.49	2.76
		2,000	5	0.33	0.18	0.22	0.42	0.35	0.20	0.23	0.44	0.40	0.22	0.26	0.48
			10	3.45	1.86	1.76	3.24	3.22	1.74	1.62	2.99	2.96	1.61	1.49	2.73
	GRTM	1,000	5	0.27	0.19	0.15	0.16	0.33	0.18	0.24	0.26	0.22	0.16	0.16	0.20
			10	0.35	0.20	0.25	0.44	0.36	0.21	0.25	0.45	0.40	0.23	0.27	0.49
		2,000	5	0.27	0.17	0.14	0.12	0.18	0.14	0.12	0.12	0.19	0.14	0.13	0.16
			10	0.35	0.18	0.24	0.41	0.37	0.20	0.24	0.44	0.41	0.23	0.25	0.47
*Bias*	GRM	1,000	5	0.22	0.02	−0.15	−0.35	0.23	0.02	0.15	−0.37	0.30	0.04	−0.16	−0.41
			10	2.42	0.94	−0.63	−2.03	2.66	0.86	0.58	−2.18	2.89	0.78	−0.54	−2.39
		2,000	5	0.20	0.01	−0.14	−0.29	0.21	0.01	0.13	−0.36	0.27	0.02	−0.14	−0.38
			10	2.40	0.92	−0.67	−2.05	2.64	0.87	−0.60	−2.16	2.84	0.76	−0.54	−2.36
	GRTM	1,000	5	−0.17	−0.11	−0.07	−0.01	−0.13	−0.10	−0.07	−0.05	−0.08	−0.09	−0.10	−0.11
			10	0.23	0.02	−0.15	−0.36	0.24	0.02	−0.16	−0.36	0.29	0.04	−0.19	−0.43
		2,000	5	−0.15	−0.09	−0.07	−0.01	−0.14	−0.11	−0.09	−0.05	−0.07	−0.09	−0.09	−0.10
			10	0.22	0.02	−0.14	−0.33	0.23	0.03	−0.15	−0.36	0.27	0.05	−0.16	−0.40
*SEE*	GRM	1,000	5	0.27	0.20	0.19	0.25	0.28	0.21	0.20	0.26	0.28	0.23	0.23	0.27
			10	2.51	1.64	1.62	2.50	1.80	1.52	1.50	2.03	0.77	1.41	1.39	1.38
		2,000	5	0.19	0.06	0.17	0.40	0.30	0.14	0.20	0.42	0.35	0.17	0.23	0.45
			10	2.48	1.62	1.63	2.51	1.84	1.51	1.50	2.07	0.83	1.42	1.39	1.37
	GRTM	1,000	5	0.21	0.15	0.13	0.16	0.30	0.15	0.23	0.26	0.20	0.13	0.12	0.17
			10	0.26	0.20	0.20	0.25	0.27	0.21	0.19	0.27	0.28	0.23	0.19	0.23
		2,000	5	0.22	0.14	0.12	0.12	0.11	0.09	0.08	0.11	0.18	0.11	0.09	0.12
			10	0.27	0.18	0.19	0.24	0.29	0.20	0.19	0.25	0.31	0.22	0.19	0.25

For the ability parameter, as shown in Table 7, the parameter was underestimated under different conditions as indicated by the bias. The same findings for the dichotomous testlet item applied to the polytomous testlet item, as evidenced by the results that a short length of testlet and a small testlet effect were associated with a more precise estimation of the ability parameter. Additionally, a large sample size resulted in a smaller bias, SEE and RMSE for both GRM and GRTM.

**TABLE 7:** Statistical summary of the ability parameter for the polytomous testlet items.

Evaluation criteria	Model	Sample size	Length of testlet = 5			Length of testlet = 10		
			Testlet effect					
			0.5	1	2	0.5	1	2
*RMSE*	GRM	1,000	0.22	0.25	0.32	0.25	0.30	0.37
		2,000	0.21	0.24	0.28	0.24	0.29	0.32
	GRTM	1,000	0.22	0.22	0.23	0.22	0.24	0.24
		2,000	0.20	0.21	0.23	0.21	0.22	0.23
*Bias*	GRM	1,000	−0.08	−0.11	−0.14	−0.10	−0.13	−0.19
		2,000	−0.07	−0.09	−0.13	−0.09	−0.13	−0.17
	GRTM	1,000	−0.09	−0.08	−0.08	−0.09	−0.08	−0.09
		2,000	−0.08	−0.06	−0.07	−0.07	−0.07	−0.09
*SEE*	GRM	1,000	0.20	0.22	0.29	0.23	0.27	0.32
		2,000	0.20	0.22	0.25	0.22	0.26	0.27
	GRTM	1,000	0.20	0.20	0.22	0.20	0.23	0.22
		2,000	0.18	0.20	0.22	0.20	0.21	0.21

## Discussion

In this study, we compared the performance of IRT models and TRT models for the dichotomous and polytomous items in the context of equating tests composed of testlets. For achieving the most generalization, in this study, the 2PL and the TRT model were selected as the item response functions for the dichotomous items, and the GRM and GRTM model were selected as the item response functions for the polytomous items. In addition, several factors were examined through the simulation studies including (a) testlet effect, (b) length of testlet items, and (c) sample size.

The simulation results showed that the TRT model always performed much more better than 2PL model when LID was present across all the test conditions. Previous studies had demonstrated that the TRT model could provide more flexibility and accuracy to the testlet-based test equating (Bradlow et al., 1999; Wainer et al., 2000; DeMars, 2006; Cao et al., 2014). Further, in addition to the confirmation of previous findings, one important contribution of this study was that a comparison was made between GRM and GRTM, which was an extension of testlet-based equating to the polytomous testlet response theory model. Despite the growing recognition of the testlet-based equating, the polytomous testlet response theory model has received little attention in the literature. Comparisons made in this study showed that the GRTM yielded more accurate item parameter estimates than the GRM when LID was present. One possible explanation could be that the GRTM, as a development from the GRM, provides more accuracy to model testlet-based tests. Therefore, use of the TRT-based models is recommended for both the dichotomous and polytomous items as they will minimize the impact of LID on the testlet-based equating.

Moreover, as in the simulation study, several factors were examined. In terms of testlet effect, it was seen that both the 2PL model and GRM were more sensitive, whereas the TRT model and GRTM seemed relatively robust as testlet effect increased from low to high. This general pattern has been consistently observed in the previous study with the comparison of different IRT models on testlet-based test equating for the dichotomous items (Cao et al., 2014), but the previous study has not taken the polytomous items into consideration. Concerning the length of testlet items, it was clear, as discussed earlier, that the TRT model and GRTM were more accurate as the length of testlet items increased than were the 2PL model and GRM. More specifically, the 2PL model and GRM consistently revealed a substantial amount of bias in parameter estimating, which led to larger overall equating errors. This may be the case because both the 2PL model and GRM could lead to the misestimation of item parameters when they were used to handle the LID caused by testlet (Zenisky et al., 2002; Keller et al., 2003). Under the NEAT design, both IRT-based models and TRT-based models tended to have smaller errors with a larger sample size primarily due to the reduced errors of parameter estimating. Given the fact that most equating procedures require large samples for accurate estimates (Kolen and Brennan, 2014; Babcock and Hodge, 2019).

Although the current research successfully used the concurrent calibration to compare the performance of IRT models and TRT models for the dichotomous and polytomous items in the context of equating tests composed of testlets, it is not without limitations. First, there are various polytomous IRT models, such as the nominal response model, or the generalized partial credit model (Nering and Ostini, 2010; van der Linden, 2016). More research is needed to compare polytomous items with other models in the context of equating tests composed of testlets. Second, this article considered two particular test formats: dichotomous items and polytomous items, respectively. In practice, the test format (e.g., mixed-format tests) might be more complex depending on the purpose of the test (von Davier and Wilson, 2007). Future research could focus on testlet-based equating with other types of test formats. Third, careful attention should be paid to the generalization of these findings because of the specific conditions in these two simulation studies (as shown in Tables 8, 9). For example, the discrimination parameter used in our studies are higher compared with other test equating studies. Future research should continue to investigate the performance of TRT-based models in other equating contexts, such as the equating for the multidimensional tests (Kim et al., 2019).

**TABLE 8:** Parameters of the dichotomous items for the reference form and new form.

	Reference form					New form			
	Items	Testlets	a	b		Items	Testlets	a	b
Non-anchor items	1		1.53	0.18	Non-anchor items	1		1.45	−0.59
	2		2.32	−1.49		2		1.04	−0.17
	3		1.04	−1.62		3		1.83	−1.19
	4		1.71	0.89		4		1.25	−1.62
	5		1.47	0.63		5		1.40	1.63
	6		1.68	−1.22		6		1.52	−1.22
	7		1.49	−0.07		7		1.42	−1.47
	8		1.48	−0.04		8		1.53	−0.51
	9		2.20	−1.05		9		1.92	1.68
	10		1.13	−2.05		10		1.64	0.55
	11		1.04	−0.56		11		1.37	−0.34
	12		1.11	−1.30		12		1.56	1.67
	13		1.28	0.78		13		1.46	0.85
	14		1.53	0.91		14		1.58	0.32
	15		1.35	0.67		15		1.12	0.17
	16		1.68	−1.58		16		1.62	−0.03
	17		1.73	0.43		17		1.94	0.20
	18		1.64	0.65		18		1.02	0.12
	19		1.11	0.15		19		1.53	0.50
	20		1.85	−0.57		20		1.23	−0.93
	21	1	1.09	1.35		21	1	1.52	1.64
	22	1	2.32	1.53		22	1	1.67	−1.11
	23	1	1.52	0.97		23	1	1.68	−1.11
	24	1	0.81	0.54		24	1	1.70	−0.71
	25	1	1.25	1.05		25	1	0.24	0.19
	26	2	1.39	0.01		26	1	1.12	−0.45
	27	2	1.91	−1.43		27	1	1.43	0.62
	28	2	1.48	1.76		28	1	0.68	−0.40
	29	2	2.36	−0.44		29	1	1.68	0.72
	30	2	1.76	−0.61		30	1	2.34	−1.86
	31	3	1.04	−1.05		31	2	1.74	−0.83
	32	3	2.15	−0.86		32	2	1.70	0.65
	33	3	1.75	0.24		33	2	0.80	0.81
	34	3	1.35	−0.55		34	2	1.75	0.27
	35	3	1.78	0.66		35	2	1.62	−0.19
	36	4	1.91	−0.99		36	2	1.72	0.87
	37	4	1.63	2.87		37	2	0.31	−1.49
	38	4	1.12	−0.07		38	2	0.96	−0.38
	39	4	1.23	−0.68		39	2	1.24	0.02
	40	4	0.75	0.11		40	2	1.78	0.17
	
Anchor items	41	5	1.45	−0.59	Anchor items	41	3	1.46	−0.15
	42	5	1.04	−0.17		42	3	1.51	−0.64
	43	5	1.83	−1.19		43	3	1.87	1.10
	44	5	1.25	−1.62		44	3	1.67	0.27
	45	5	1.40	1.63		45	3	1.53	−0.43
	46	6	1.52	−1.22		46	3	1.24	0.40
	47	6	1.42	−1.47		47	3	1.52	−1.53
	48	6	1.53	−0.51		48	3	1.52	−0.52
	49	6	1.92	1.68		49	3	1.69	0.01
	50	6	1.64	0.55		50	3	1.82	1.31
	51		1.37	−0.34		51		1.56	−1.62
	52		1.56	0.61		52		1.46	−0.06
	53		1.46	0.85		53		1.47	−0.35
	54		1.58	0.32		54		1.61	0.86
	55		1.12	0.17		55		1.42	−0.73
	56		1.62	−0.03		56		1.78	−0.84
	57		1.94	0.20		57		2.00	−0.83
	58		1.02	0.12		58		2.14	0.42
	59		1.53	0.50		59		1.56	−0.39
	60		1.23	−0.93		60		1.80	−1.70
Mean			1.51	−0.06				1.50	−0.12
Standard deviation			0.36	1.00				0.38	0.92

**TABLE 9:** Parameters of the polytomous items for the reference form and new form.

Reference form								New form							
	Items	Testlets	a	b1	b2	b3	b4		Items	Testlets	a	b1	b2	b3	b4
Non-anchor items	1		0.59	−0.03	0.27	0.50	0.56	Non-anchor items	1		1.21	−1.40	−1.17	−0.24	0.80
	2		0.59	−2.45	−1.52	−0.96	0.10		2		1.12	−2.00	−0.72	0.05	0.06
	3		1.39	−1.38	−0.17	0.47	1.73		3		1.13	−1.02	−0.61	−0.35	0.15
	4		1.20	−0.70	0.35	0.77	1.01		4		1.18	−1.07	−0.86	−0.19	0.44
	5		1.31	−0.20	0.15	0.63	2.07		5		1.27	−1.17	−0.76	−0.20	1.60
	6		0.88	−0.49	0.00	0.36	1.19		6		1.37	−0.67	−0.66	−0.35	0.18
	7		0.83	−1.01	−0.46	−0.30	0.70		7		1.21	−2.63	−0.53	1.35	2.56
	8		0.68	−1.00	0.46	0.98	1.35		8		1.21	−1.10	0.04	0.42	0.44
	9		1.17	−0.32	0.00	0.23	1.71		9		1.01	−0.57	0.32	0.50	1.23
	10		1.36	−0.93	−0.76	−0.52	1.04		10		1.46	0.13	0.90	0.96	1.01
	11		1.28	−1.68	−0.99	−0.48	1.33		11		1.11	−0.01	0.92	1.58	2.47
	12		0.74	−1.17	−0.73	−0.69	0.76		12		1.31	−0.92	−0.68	0.43	0.76
	13		1.26	−1.34	−1.15	0.22	0.52		13		1.59	−1.12	−1.09	−0.52	0.21
	14		1.00	−0.58	−0.05	0.29	1.20		14		1.74	0.22	0.37	1.14	1.14
	15		1.37	−0.36	−0.09	0.81	1.02		15		1.18	−0.15	0.54	0.86	0.92
	16		0.74	−1.86	−0.21	0.12	1.26		16		1.62	−0.53	−0.07	0.07	0.64
	17		0.80	−0.89	−0.32	0.37	0.73		17		1.20	−0.47	0.15	0.85	1.50
	18		0.74	−1.67	−0.72	0.62	1.32		18		1.22	−0.89	−0.21	0.52	0.66
	19		1.31	−0.92	−0.65	0.41	1.66		19		1.18	−0.90	−0.84	−0.75	0.75
	20		0.86	−1.04	−0.78	−0.15	1.28		20		1.46	−1.62	−0.83	0.12	1.22
	21	1	1.00	−0.59	0.16	0.20	0.64		21	1	0.98	−1.18	−0.93	−0.66	1.25
	22	1	1.60	−0.79	−0.09	0.05	1.46		22	1	1.06	−0.35	−0.19	0.37	0.87
	23	1	1.03	−1.48	−0.64	−0.46	0.46		23	1	0.35	−0.87	−0.42	0.42	0.98
	24	1	0.59	−0.40	1.22	1.23	2.06		24	1	0.79	−1.42	−0.90	−0.47	0.49
	25	1	0.89	−0.88	−0.40	1.03	2.53		25	1	1.11	−0.30	−0.16	−0.16	0.61
	26	2	1.00	−1.29	−0.94	−0.38	0.77		26	1	1.29	−1.28	−0.97	−0.85	0.74
	27	2	1.24	−2.03	−1.34	−0.05	0.59		27	1	1.32	−0.87	−0.31	0.50	1.01
	28	2	1.13	−0.88	−0.47	0.07	1.06		28	1	1.44	−1.04	0.27	0.71	1.54
	29	2	0.60	−0.97	−0.67	−0.03	0.71		29	1	0.47	−1.36	−0.81	−0.37	−0.16
	30	2	0.97	−0.62	0.01	0.94	1.59		30	1	1.11	0.13	0.41	1.10	1.32
	31	3	1.23	−1.20	−1.01	0.64	0.80		31	2	0.71	−0.88	−0.03	0.06	0.42
	32	3	1.07	−0.36	−0.34	−0.32	0.54		32	2	0.85	−0.95	0.26	2.02	2.54
	33	3	0.36	−1.04	−0.49	0.14	1.00		33	2	1.18	−1.08	−0.85	−0.07	0.56
	34	3	0.76	−1.68	0.69	0.74	1.01		34	2	1.08	−1.17	0.30	0.98	1.70
	35	3	0.90	0.45	0.72	1.16	1.69		35	2	1.15	−1.24	−0.40	0.91	1.55
	36	4	1.80	−1.81	0.14	0.96	1.19		36	2	0.46	−0.70	−0.18	0.52	0.84
	37	4	1.13	0.66	0.88	1.01	1.93		37	2	0.55	−2.01	−0.78	−0.38	−0.10
	38	4	1.26	−1.20	−0.15	0.44	0.79		38	2	1.68	−1.45	−0.31	−0.22	0.22
	39	4	1.00	−2.07	0.09	0.11	0.19		39	2	0.90	−2.05	−0.59	1.51	1.60
	40	4	0.56	−1.26	−0.38	0.23	0.62		40	2	1.98	−0.09	0.99	1.98	2.42
	
Anchor items	41	5	1.21	−1.40	−1.17	−0.24	0.80	Anchor items	41	3	0.93	−1.19	−1.14	−0.15	0.23
	42	5	1.12	−2.00	−0.72	0.05	0.06		42	3	1.57	−0.01	0.20	1.20	1.26
	43	5	1.13	−1.02	−0.61	−0.35	0.15		43	3	0.86	−2.01	0.25	0.75	1.07
	44	5	1.18	−1.07	−0.86	−0.19	0.44		44	3	0.69	−0.33	0.79	0.96	1.07
	45	5	1.27	−1.17	−0.76	−0.20	1.60		45	3	1.22	−0.94	−0.27	0.68	0.77
	46	6	1.37	−0.67	−0.66	−0.35	0.18		46	3	0.89	−0.94	−0.68	0.48	2.29
	47	6	1.21	−2.63	−0.53	1.35	2.56		47	3	0.80	−1.19	−1.11	−0.40	0.19
	48	6	1.21	−1.10	0.04	0.42	0.44		48	3	1.30	−1.50	−1.03	−0.21	0.71
	49	6	1.01	−0.57	0.32	0.50	1.23		49	3	1.03	−0.39	0.28	0.29	0.35
	50	6	1.46	0.13	0.90	0.96	1.01		50	3	1.11	−2.57	−1.05	−0.52	1.02
	51		1.11	−0.01	0.92	1.58	2.47		51		0.94	−1.57	0.05	0.51	0.90
	52		1.31	−0.92	−0.68	0.43	0.76		52		0.64	−2.00	0.75	0.79	1.30
	53		1.59	−1.12	−1.09	−0.52	0.21		53		1.07	−0.61	−0.48	1.65	2.36
	54		1.74	0.22	0.37	1.14	1.14		54		0.67	−0.47	0.00	0.10	0.35
	55		1.18	−0.15	0.54	0.86	0.92		55		1.36	0.52	0.59	1.17	1.24
	56		1.62	−0.53	−0.07	0.07	0.64		56		0.97	−0.27	0.00	0.30	0.40
	57		1.20	−0.47	0.15	0.85	1.50		57		0.68	−2.02	−0.25	0.51	1.23
	58		1.22	−0.89	−0.21	0.52	0.66		58		0.54	−0.69	−0.23	0.03	1.01
	59		1.18	−0.90	−0.84	−0.75	0.75		59		1.01	−1.74	−1.33	−1.30	0.54
	60		1.46	−1.62	−0.83	0.12	1.22		60		0.70	−1.95	0.62	0.67	0.84
Mean			1.10	−0.96	−0.27	0.29	1.05				1.10	−1.00	−0.26	0.36	0.97
SD			0.31	0.67	0.61	0.58	0.60				0.33	0.69	0.61	0.72	0.66

## Data Availability Statement

The raw data supporting the conclusion of this article will be made available by the authors, without undue reservation.

## Author Contributions

FH designed the study, conducted the simulation study, and drafted the manuscript. ZL participated in designing the study and conducted the simulation study. JS conducted the literature review. YL and LY conducted the data analysis. MZ revised the manuscript. All authors read and approved the final manuscript.

## Conflict of Interest

The authors declare that the research was conducted in the absence of any commercial or financial relationships that could be construed as a potential conflict of interest.

## Publisher’s Note

All claims expressed in this article are solely those of the authors and do not necessarily represent those of their affiliated organizations, or those of the publisher, the editors and the reviewers. Any product that may be evaluated in this article, or claim that may be made by its manufacturer, is not guaranteed or endorsed by the publisher.
