# Supplementary material for: An Extension of Testlet-Based Equating to the Polytomous Testlet Response Theory Model
Source: Front Psychol. 2022 Jan 12;12:743362. doi: 10.3389/fpsyg.2021.743362 (PMC8790509; doi:10.3389/fpsyg.2021.743362)
Supplement: Supplementary file 1 [file Data_Sheet_1.docx]

# Appendix

**FlexMIRT (e.g.: the sample size was 2000, the length of testlet was 5 and the model was 2PLM)** ：

<Project>

Title = "2PL-IRT-5";

Description = " 2PL-IRT 1 Group";

<Options>

Mode = Calibration;

Quadrature = 21,5.0;

Etol = 1e-3;

Processors = 4;

NewThreadModel = Yes;

Progress = Yes;

SaveSCO = Yes;

SavePRM = Yes;

SlopeThreshold = Yes;

Score = EAP;

<Groups>

%Graded1%

File = "U.dat";

Missing = 9;

Varnames = v1-v100;

N = 2000;

Ncats(v1-v100) = 2;

Model(v1-v100) = Graded(2);

<Constraints>

**FlexMIRT (e.g.: the sample size was 2000, the length of testlet was 5 and the model was 2PTM)** ：

<Project>

Title = "2PL-TRT-5";

Description = " 2PL-TRT 1 Group";

<Options>

Mode = Calibration;

Quadrature = 21,5.0;

Etol = 1e-3;

Processors = 4;

NewThreadModel = Yes;

Score = EAP;

Progress = Yes;

SavePRM = Yes;

SaveSCO = Yes;

<Groups>

%G%

File = "U.dat";

Varnames = v1-v100;

Missing = 9;

N = 2000;

Ncats(v1-v100) = 2;

Model(v1-v100) = Graded(2);

Dimensions = 11;

Primary = 1;

<Constraints>

Fix (v1-v100),Slope;

Free (v1-v100),Slope(1);

Free (v1-v5),Slope(2);

Free (v6-v10),Slope(3);

Free (v11-v15),Slope(4);

Free (v16-v20),Slope(5);

Free (v41-v45),Slope(6);

Free (v46-v50),Slope(7);

Free (v61-v65),Slope(8);

Free (v66-v70),Slope(9);

Free (v71-v75),Slope(10);

Free (v76-v80),Slope(11);

Equal G,(v1-v5),Slope(1) : G,(v1-v5),Slope(2);

Equal G,(v6-v10),Slope(1) : G,(v6-v10),Slope(3);

Equal G,(v11-v15),Slope(1) : G,(v11-v15),Slope(4);

Equal G,(v16-v20),Slope(1) : G,(v16-v20),Slope(5);

Equal G,(v41-v45),Slope(1) : G,(v41-v45),Slope(6);

Equal G,(v46-v50),Slope(1) : G,(v46-v50),Slope(7);

Equal G,(v61-v65),Slope(1) : G,(v61-v65),Slope(8);

Equal G,(v66-v70),Slope(1) : G,(v66-v70),Slope(9);

Equal G,(v71-v75),Slope(1) : G,(v71-v75),Slope(10);

Equal G,(v76-v80),Slope(1) : G,(v76-v80),Slope(11);

Free Cov(2,2);

Free Cov(3,3);

Free Cov(4,4);

Free Cov(5,5);

Free Cov(6,6);

Free Cov(7,7);

Free Cov(8,8);

Free Cov(9,9);

Free Cov(10,10);

Free Cov(11,11);

**FlexMIRT (e.g.: the sample size was 2000, the length of testlet was 5 and the model was GRM)** ：

<Project>

Title = "GRM-5";

Description = " GRM 1 Group";

<Options>

Mode = Calibration;

Quadrature = 21,5.0;

Etol = 1e-3;

Processors = 8;

NewThreadModel = Yes;

Progress = Yes;

SaveSCO = Yes;

SavePRM = Yes;

SlopeThreshold = Yes;

Score = EAP;

<Groups>

%Group1%

File = "U.dat";

Missing = 9;

Varnames = v1-v100;

N = 2000;

Ncats(v1-v100) = 5;

Model(v1-v100) = Graded(5);

<Constraints>

**FlexMIRT (e.g.: the sample size was 2000, the length of testlet was 5 and the model was GRTM)** ：

<Project>

Title = "GRTM-5";

Description = " GRTM 1 Group";

<Options>

Mode = Calibration;

Quadrature = 21,5.0;

Etol = 1e-3;

Processors = 8;

NewThreadModel = Yes;

Score = EAP;

Progress = Yes;

SaveSCO = Yes;

SavePRM = Yes;

<Groups>

%G%

File = "U.dat";

Missing = 9;

Varnames = v1-v100;

N = 2000;

Ncats(v1-v100) = 5;

Model(v1-v100) = Graded(5);

Dimensions = 11;

Primary = 1;

<Constraints>

Fix(v1-v100),Slope;

Free(v1-v100),Slope(1);

Free(v1-v5),Slope(2);

Free(v6-v10),Slope(3);

Free(v11-v15),Slope(4);

Free(v16-v20),Slope(5);

Free(v41-v45),Slope(6);

Free(v46-v50),Slope(7);

Free(v61-v65),Slope(8);

Free(v66-v70),Slope(9);

Free(v71-v75),Slope(10);

Free(v76-v80),Slope(11);

Equal G,(v1-v5),Slope(1) : G,(v1-v5),Slope(2);

Equal G,(v6-v10),Slope(1): G,(v6-v10),Slope(3);

Equal G,(v11-v15),Slope(1): G,(v11-v15),Slope(4);

Equal G,(v16-v20),Slope(1): G,(v16-v20),Slope(5);

Equal G,(v41-v45),Slope(1): G,(v41-v45),Slope(6);

Equal G,(v46-v50),Slope(1): G,(v46-v50),Slope(7);

Equal G,(v61-v65),Slope(1): G,(v61-v65),Slope(8);

Equal G,(v66-v70),Slope(1): G,(v66-v70),Slope(9);

Equal G,(v71-v75),Slope(1): G,(v71-v75),Slope(10);

Equal G,(v76-v80),Slope(1): G,(v76-v80),Slope(11);

Free Cov(2,2);

Free Cov(3,3);

Free Cov(4,4);

Free Cov(5,5);

Free Cov(6,6);

Free Cov(7,7);

Free Cov(8,8);

Free Cov(9,9);

Free Cov(10,10);

Free Cov(11,11)
